# Monitoring dynamics of human adenovirus disassembly induced by mechanical fatigue

**DOI:** 10.1038/srep01434

**Published:** 2013-03-13

**Authors:** A. Ortega-Esteban, A. J. Pérez-Berná, R. Menéndez-Conejero, S. J. Flint, C. San Martín, P. J. de Pablo

**Affiliations:** 1 Departamento de Física de la Materia Condensada, Universidad Autónoma de Madrid, 28049 Madrid, Spain; 2 Department of Macromolecular Structure, Centro Nacional de Biotecnología (CNB-CSIC). Darwin 3, 28049 Madrid, Spain; 3 Department of Molecular Biology, Princeton University, Princeton, NJ 08544, USA

## Abstract

The standard pathway for virus infection of eukaryotic cells requires disassembly of the viral shell to facilitate release of the viral genome into the host cell. Here we use mechanical fatigue, well below rupture strength, to induce stepwise disruption of individual human adenovirus particles under physiological conditions, and simultaneously monitor disassembly in real time. Our data show the sequence of dismantling events in individual mature (infectious) and immature (noninfectious) virions, starting with consecutive release of vertex structures followed by capsid cracking and core exposure. Further, our experiments demonstrate that vertex resilience depends inextricably on maturation, and establish the relevance of penton vacancies as seeding loci for virus shell disruption. The mechanical fatigue disruption route recapitulates the adenovirus disassembly pathway *in vivo*, as well as the stability differences between mature and immature virions.

Viruses^1^ have to deliver their genome into host cells. Many bacteriophage inject their nucleic acid genomes into bacteria leaving their capsids behind. Eukaryotic viruses are engulfed by the host cell and undergo controlled disassembly (uncoating) until their replication compartment is reached^2^,^3^. Signals received upon entering the host cell trigger a cascade of conformational changes in the proteins forming the viral shell, resulting in weakening and further disruption of the virion to expose its genome to the cellular machinery^2^,^4^. Different physicochemical agents may induce similar conformational changes in proteins composing viral shells, which ultimately surpass^5^ the activation energy of disassembly (Fig. 1a). For instance, in poliovirus the same disassembly mechanism is induced by either binding to an immunoglobulin-like receptor on the cell surface (*in vivo*) or exposure to moderate heat (*in vitro*)^6^. Investigations into virus structural stability usually rely on bulk physicochemical procedures accompanied by Electron or Atomic Force Microscopy (AFM)^7^,^8^,^9^. These imaging techniques convey static snapshots lacking real time information about the dynamics of disassembly^10^. AFM nanoindentations beyond capsid rupture strength^11^,^12^,^13^,^14^,^15^,^16^,^17^,^18^ induce mechanical failure of viruses, but likewise provide only a final image of the disruption process.

Adenovirus, a human pathogen and a potential therapeutic tool^19^, has an icosahedral shell enclosing the dsDNA genome associated with a large amount of protein, to form the viral core^20^,^21^,^22^ (Fig. 1b). Adenovirus uncoating in the cell occurs in a stepwise manner^23^, beginning at the plasma membrane where receptor binding induces loss of protruding fibers^24^. Virion dismantling continues in the early endosome, where mild acidification causes release of a few vertex capsomers (pentons) and peripheral core components^10^,^25^,^26^. The partially disrupted particle escapes the endosome and travels to the nuclear pore, where final disassembly occurs and the genome enters the nucleus^27^ (Fig. S4 in Supporting Information -SI-). To achieve its full infectious potential, adenovirus requires a maturation process during which a viral protease cleaves several capsid and core proteins, making the virions metastable and primed for sequential uncoating^10^. A mutant called *ts1* does not undergo the proteolytic cleavages and is stalled at the immature state (Fig. 1b, *ts1*)^28^. The presence of uncleaved precursor proteins makes *ts1* virions more stable than wild type (WT) (Fig. 1b, WT), impairing proper uncoating and aborting infection^10^,^29^. Unexpectedly, AFM nanoindentation assays revealed that the extra stability of *ts1* does not equate with greater stiffness^10^, and failed to show differences in breakage pattern between mature and immature adenovirus that could explain the different uncoating behaviour (SI and Fig. S1).

## Results

**Figure 1:**
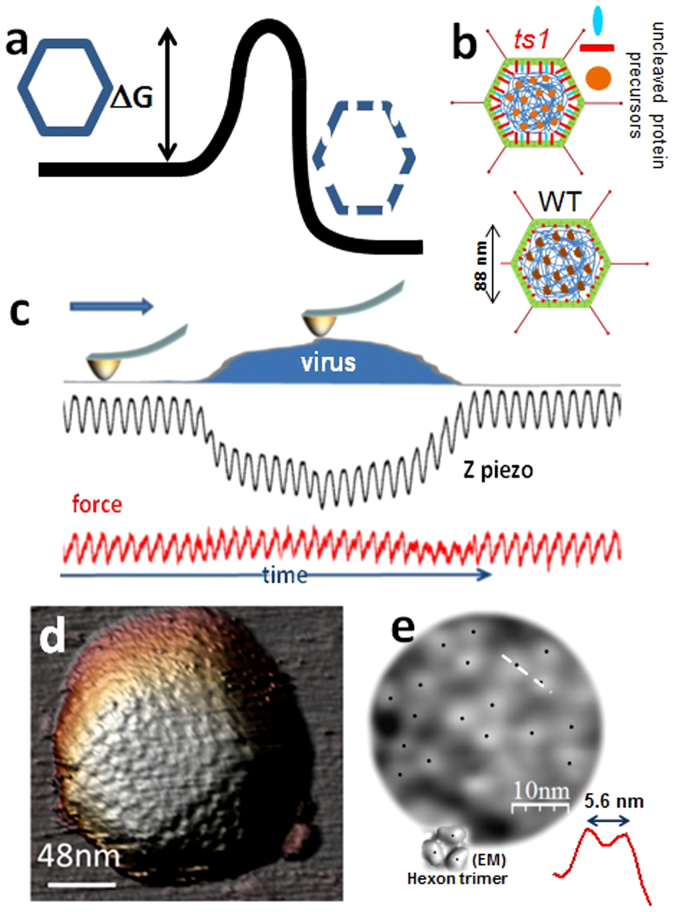
Adenovirus maturation and AFM imaging. (a) Sketch representing the energetics of viral disassembly. (b) Schematic representation of *ts1* (immature) and WT (mature) adenovirus structure. (c) Cartoon depicting the Jumping Mode imaging force cycles (red), as well as the z-piezo actuation (black) along a single scan-line. (d) AFM topography of adenovirus in liquids, presenting a view along the 3-fold symmetry axis. (e) High pass filtered version of the central triangular facet of (d) revealing the towers of the triangular hexons cued by black dots. The red topographical profile inset is taken along the white dashed line of (e). An inset showing a hexon trimer as imaged by cryo-electron microscopy^36^ is shown with black dots indicating each tower for comparison.

Here we show how mechanical fatigue acts as a stress agent that induces gradual capsid disruption. Adenovirus particles adsorbed to mica (SI) were imaged in buffer solution by AFM scanning in Jumping Mode^30^,^31^, which requires performing consecutive loading cycles of a few hundred pN (Fig. 1c). Figures 1d and S2 present typical AFM images of adenovirus capsids, visualized along the 3-fold symmetry axis. High pass filtering resolves the protruding towers of the trimeric capsomers (hexons) in the icosahedral facet (Fig. 1e), indicating a lateral resolution better than 3 nm. When we acquired successive images of the same virion at ~100 pN, well below the capsid rupture force^10^, these repeated loading series induced mechanical fatigue^32^ simultaneously triggering and enabling real time monitoring of adenovirus disassembly.

**Figure 2:**
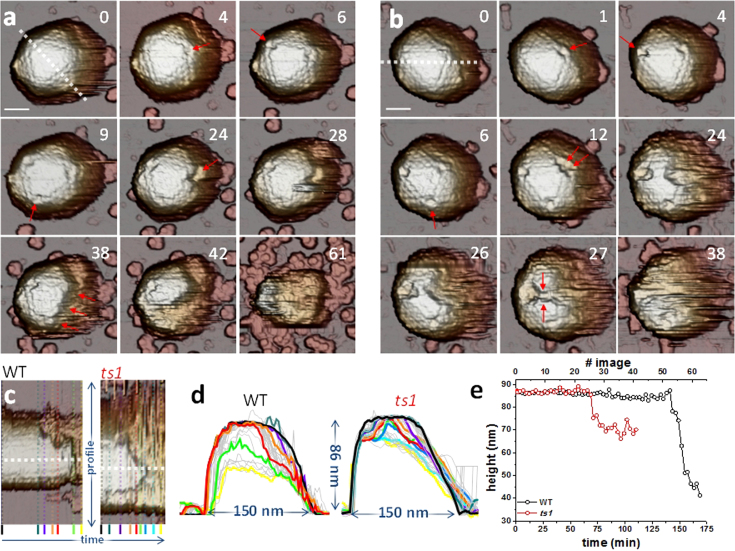
Mature and immature adenovirus disruption by material fatigue. (a and b) Selected individual frames along the disassembly process of one WT (a) and one *ts1* (b) virion. Movies S1 and S3 of SI show complete image datasets for these two viruses. Penton vacancies are highlighted with arrows. The numbers refer to the position of the frame on the corresponding movie. In (a), frame 38, the arrows indicate the crumbling direction. In (b), arrows in frame 27 indicate coalescence of voids. Scale bars correspond to 46 nm and 40 nm for WT and *ts1*, respectively. (c) Kymographs of the profiles of WT and *ts1*, as indicated, corresponding to the white dashed lines in frames #0 of (a) and (b). (d) Transversal profiles obtained from (c) showing the shape evolution of WT and *ts1* virus. Colored curves denote profiles corresponding to same colored profiles in (c). The remaining profiles are depicted in grey. (e) Evolution of the maximum height of the WT and *ts1* particles along time, obtained at the white dotted profiles in (c).

Figure 2 exemplifies the results obtained for mature (WT, Fig. 2a) and immature (*ts1*, Fig. 2b) particles. The mature virus was scanned 61 times during 174 minutes, and a movie was generated from the consecutive images (SI, movie S1). Frame 0 in Fig. 2a presents the intact particle of ~86 nm height (Fig. 2d, black), in agreement with the nominal diameter of 88 nm from facet to facet. Frames 4 to 9 reveal that pentons are sequentially lost from each vertex of the triangular facet. The upper right penton vacancy then seeds a growing fracture (frame 24) that evolves into crumbling of the particle from right to left (frames 28 to 42, movie S1), finishing in a blob which lacks the whole shell and most of the core (frame 61). From these images, a kymograph was generated (Fig. 2c) consisting of the topographical profiles, plotted in Fig. 2d, of all frames along the dotted white line indicated in frame 0, enabling their comparison along time. WT height decreased continuously from 86 nm (frame 0, black) to 35 nm (frame 61, yellow). In fact, profiles purple (frame 28), orange (frame 38) and red (frame 42) reveal that at the locations where the shell appears intact the height remains unchanged, while at the broken parts the AFM tip reaches deeper and deeper, releasing the core as soon as the protein cage is broken. This observation is further supported by the evolution of particle height along time (Fig. 2e, black) provided by the horizontal white dotted profile of Fig. 2c: up to frame 51, the height drops by only a few nm; but it decreases sharply by ~45 nm in only 6 images, indicating that the virion contents are quickly released concurrently with crumbling of the virus shell.

The *ts1* (immature) adenovirus particle in Fig. 2b observed in ~40 images taken along 118 minutes disassembled in a different manner than WT. Here pentons sequentially pop off at frames 1, 4 and 6 (SI, movie S3). From the two penton vacancies at the upper vertices of the triangular facet, two simultaneous voids grow (frames 24 and 26) and coalesce (frame 27). Comparison of the consecutive profiles obtained from Fig. 2c (*ts1*) reveals that while the void areas keep growing, their depth soon stops increasing (dark blue and cyan profiles in Fig. 2d, *ts1*). The plot showing height evolution along the white dotted line in Fig. 2c (Fig. 2e, red), indicates that it stays constant until frame 23, when it undergoes a sharp decrease of ~16 nm. Afterwards the height remains constant at 70 nm, revealing a stable structure after the virus shell has been removed.

**Figure 3:**
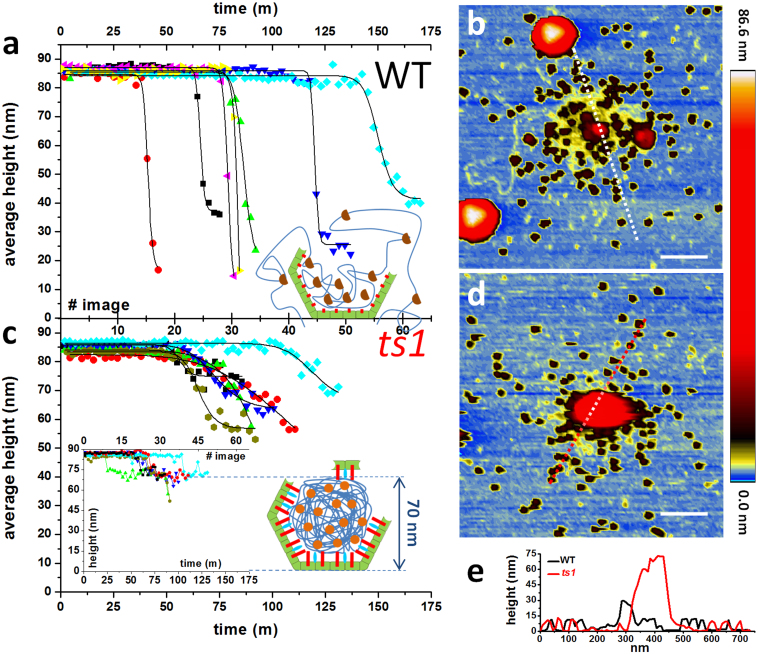
Topographical evolution along time. (a and c) Evolution of the average height of 7 WT (a) and 6 *ts1* particles (c) along time. Solid curves are the sigmoidal fits of the data (table S1). The inset chart in (c) depicts the maximum height evolution along time for *ts1*. Inset cartoons show models of particle dismantling. (b and d) Optimized color palette to enhance substrate details in topographical images after fatigue experiment. (e) Profiles corresponding to the dashed lines of (b) and (d). Scale bars correspond to 200 nm.

In contrast to classical nanoindentation studies^33^, mechanical fatigue experiments require not only intact particles, but also stable attachment to the surface, during several hours of repeated scanning of a single particle in liquid. Accordingly, only viral particles adsorbed on 3-fold symmetry orientations were stable enough, presumably because this geometry maximizes the virus-surface area of contact. Once the consecutive imaging started, about 60% of viruses detached before disassembly ended. Nevertheless, a dataset comprising 7 WT and 6 *ts1* viruses was analyzed as described above, revealing a consistent and reproducible behavior pattern. The average height (Methods) of each WT particle (Fig. 3a) remained constant until it quickly dropped to highly disperse final values. Figure 3b shows the typical topography of a mature particle after complete disassembly, demonstrating that the virus has been disrupted in two major pieces of about 30 nm in diameter. In addition, there are smaller objects (~100) whose height (~12 nm, Fig. 3e) is compatible with those of individual hexons^34^. Height measurements along time were fitted to a Hill sigmoid function 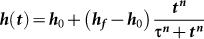, where *h_0_*, *h_f_, τ* and *n* are the initial and final heights, inflection point, and cooperative factor, respectively. The WT average cooperative coefficient for height decrease was n~77 (table S1). Conversely, the average heights of *ts1* particles (Fig. 3c) present a more gentle decrease, reflected by a lower cooperative coefficient n ~ 13 (table S1). The maximum height profile of each *ts1* particle (inset chart in Fig. 3c) underwent a sharp decrease of about 16 nm, reaching a stable value at ~70 nm. The typical topography of an immature particle after complete disassembly (Fig. 3d) shows a mostly intact core of 70 nm in height plus numerous surrounding hexons (Fig. 3e).

**Figure 4:**
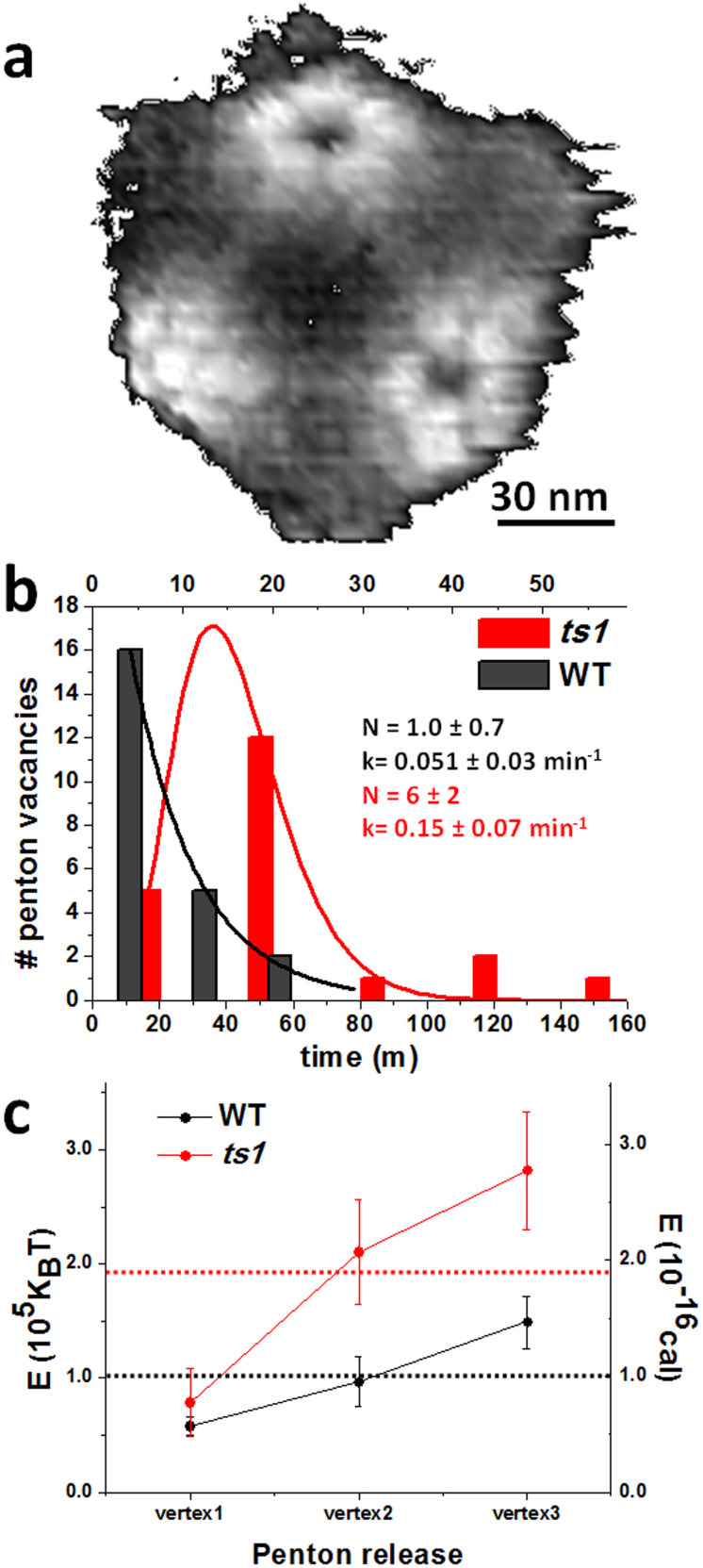
Dynamics of penton release. (a) Image of a virus lacking some pentons, high pass filtered to enhance visibility of the vertex region. (b) Histograms showing timing of penton release for all WT (black) and all *ts1* (red) viruses. The parameters of the gamma distribution fitted to the histograms (shape factor N and rate k) are indicated. (c) Plot showing the estimations of energy supplied to WT (black) and *ts1* (red) viruses for each penton vacancy creation. Red and black horizontal dotted lines indicate the average energy from the three penton vacancies for WT and *ts1*, respectively. See also Fig. S5.

Material fatigue assays provide novel quantitative information on single virus disruption, such as the dynamics of penton release (Fig. 4a and S3). Since viruses are oriented following the 3-fold symmetry, we consider the fate of the three pentons visible for each particle in the three-fold orientation, although virus tilting occasionally enables imaging of lateral pentons. The number of penton vacancies of all viruses appearing at each time (Methods) during the experiment was plotted as a histogram (Fig. 4b, table S2) and fitted to a gamma distribution 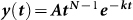, where A and N are the normalization and shape factors respectively, and k is the rate parameter^35^. The expected times for penton release according to this formulism (N/k) are ~20 minutes (7 images) for WT and ~42 minutes (15 images) for *ts1* particles. The time lag between penton loss (frame 9 of Fig. 2a) and final dismantling, defined by the frame when blurring of the icosahedral contour occurs (frame 42 of Fig. 2a), was also computed. For the WT virus in Fig. 2a, 78% of the total disassembly time elapsed between loss of the third penton and complete destruction. On average, WT particles kept their gross pentonless organization during 66 ± 8% of the total experiment, while pentonless *ts1* virions endure for 40 ± 10% of this time (table S3).

The energetics of penton release can be roughly estimated by accounting the imaging energy provided at each pixel (Methods and SI, Fig. S5). From this approximation, the average energy applied to generate one penton vacancy is 1.9 × 10^−16^ calories for *ts1* and 1.0 × 10^−16^ calories for WT (Fig. 4c), indicating that pentons in *ts1* are more stable than in WT. Interestingly, estimations from a bulk technique such as differential scanning calorimetry indicate that the energy cost of the initial disassembly events (that most likely correspond to release of one or several pentons) is 6.0 × 10^−17^ cal/virion for *ts1* and 3.4 × 10^−17^ cal/virion for WT^10^. The energy ratio *ts1*/WT from our single virion experiments is 1.9, in good agreement with the bulk calorimetric results (1.8). Both indicate that *ts1* pentons are more stable than WT ones.

**Figure 5:**
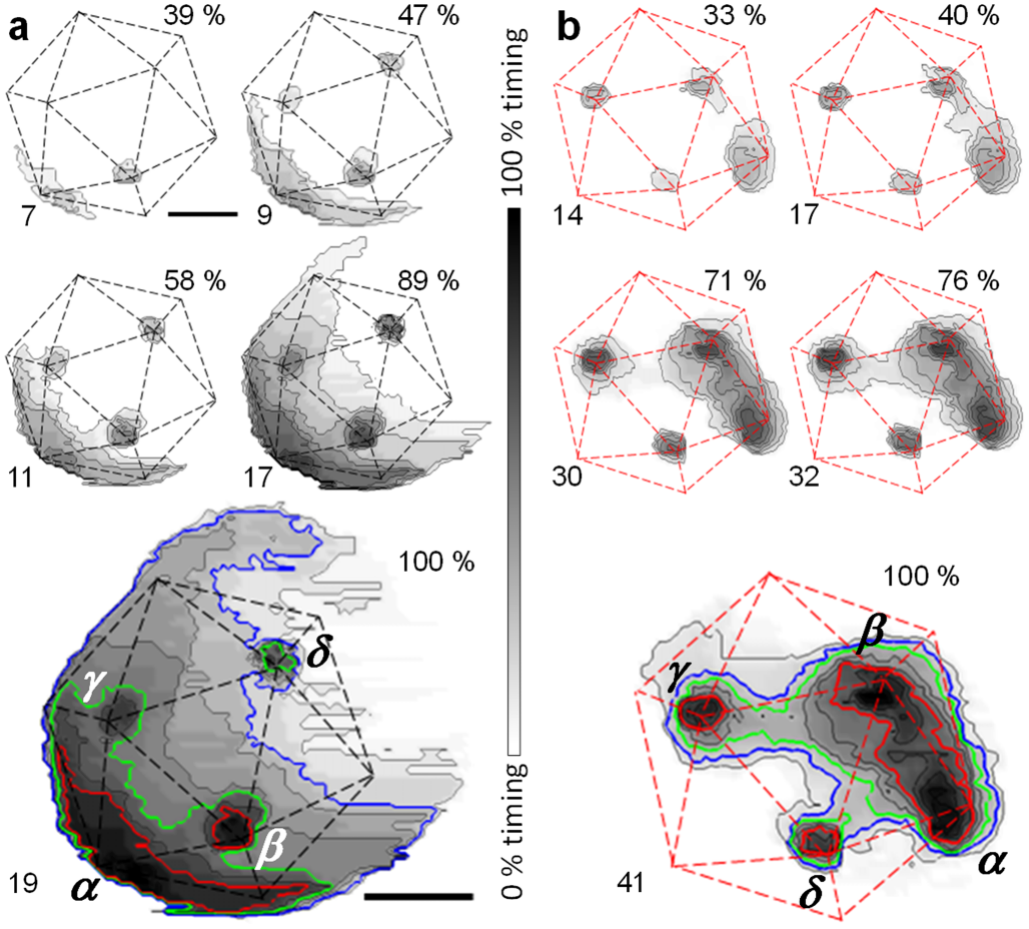
Time cumulative disruption maps (TCDMs). Disruption maps for WT (a) and *ts1* (b) virions. The elapsed time in minutes corresponding to the whole grayscale is 50 min in (a) and 118 min in (b). The scale bar corresponds to 48 and 40 nm for (a) and (b), respectively. Red contour plots indicate areas removed early (32% and 43% of the elapsed time for (a) and (b), respectively). Green contour plots indicate areas removed at medium times (55% in (a), 74% of the elapsed time in (b)). Blue contour plots indicate areas removed late (84% and 83% of the elapsed time). See also movies S1–S5 of SI and Fig. S6.

To explore the capsid rupture pattern for each particle, we calculated time cumulative disruption maps (TCDMs) depicting the sequence in which different shell areas were lost from the virion (Fig. 5 and S6). In these maps, darker grey levels indicate regions in the shell that were removed earlier during the experiment (Methods and SI). TCDMs show virus vertices as black areas because pentons are released first (Fig. 5). For example, in the mature virion in Fig. 5a, the penton vacancy created at the position denoted as *α* induces earlier material elimination than vacancies *β*, *γ* and δ (red contour line -CL- of Fig. 5a), and grows in an irregular disassembly front that reaches vertices *β* and *γ* (green CL). This crumbling spreads upwards, eventually reaching vacancy δ (blue CL, Fig 5a and movie S5). Likewise, in the TCDMs series for the immature particle in Fig. 5b, the initial CLs surrounding vertices *α* and *β* show simultaneous and independent growth spread, eventually merging into a continuous crack (red CL in Fig. 5b). Each lost vertex acts as a seed developing cracks that eventually merge with those of the neighboring vertices (*γ* and δ, green and blue CLs in Fig. 5b, movie S3).

## Discussion

Our results reveal that mechanical fatigue induces stepwise dismantling of adenovirus capsids, replicating the *in vivo* disassembly process^23^ and showing differences in the disassembly pathway for mature and immature virions that reflect their infectious phenotype. Disruption due to mechanical fatigue starts with pentons dissociating, as also occurs in response to thermal or chemical disruption^10^ and during natural disassembly at the start of the infectious cycle. However, real time monitoring shows for the first time that pentons are sequentially removed, an aspect that could not be elucidated with the techniques previously used^10^. In principle, the structure of pentons and peripentonal proteins should be identical for all the vertices, and should be equally affected by mechanical fatigue. As a consequence, they should pop off after receiving the same loading cycles. The sequential removal of pentons may indicate either uncontrolled processes of energy dissipation in the virion or subtle differences on vertex structure.

Our experiments unveil differences in the disruption dynamics of mature and immature particles. Pentons required more loading cycles to fall off in *ts1* than in WT capsids (Fig. 4b), where the creation of penton vacancies follows an exponential decay distribution consistent with a stochastic process. Moreover, the energy required to cause penton release from *ts1* particles is 1.9 times larger than for WT (Fig. 4c). The origin of the stabilization of pentons in the immature virus is twofold. First, structural studies have shown that the presence of precursor proteins results in extra interactions joining hexons (not pentons) to the core (Fig. 1b, *ts1*)^10^,^36^,^37^. Second, processing by the viral protease induces core decompaction accompanied by an increase in capsid stiffness^10^,^36^, suggesting that maturation could increase the internal pressure in the virion^10^,^17^,^38^. Both aspects may contribute to change the built-in mechanical stress at the vertices^39^ in the mature adenovirus virion that would result in more unstable pentons.

After sequential release of pentons, the capsid is peeled away to reveal the core, and finally the particle collapses. In the cell, adenovirus escapes the endosome at 15 min post infection (p.i.), and is found at the nuclear envelope at 45 min p.i., with complete disassembly occurring for the majority of particles at 60 min p.i.^23^,^40^ (Fig. S4). Therefore, viruses must remain reasonably intact in the cytosol after releasing pentons for at least 30–45 minutes. Our results indicate that WT particles keep their pentonless structure during 66 ± 8% of the disassembly elapsed time, which is similar to the *in vivo* period required for time traveling from endosome to the nuclear pore (Fig. S4).

TCDMs (Fig. 5 and S6) show that mature capsids unzip from a single fracture in the shell that starts at a penton vacancy and then spreads through the remaining shell in a highly cooperative way. Such crumbling is accompanied by simultaneous release of the material below the disappearing shell, with WT particles reaching final heights (Fig. 3a) well below the core diameter (60 nm)^36^. Topographies of mature particles after disassembly (Fig. 3b, e) reveal three kind of debris: first, large pieces of about 30 nm in height, corresponding to residual viral shell attached to the mica surface, along with core debris (red); second, tens of individual hexons dispersed around the disassembly area (black); third, partially compacted ds-DNA (yellow) that homogeneously surrounds the central area of the core debris. These results imply that the DNA and proteins forming the core are not strongly held together, or attached to the capsid walls. This property would facilitate DNA diffusion outside of the viral cage (Fig. 3a, inset) prior to transport through the nuclear pore. This infection mechanism requires certain directionality in releasing the genome since DNA has to pass through the nuclear pore. In fact, TCDMs of mature virions show that crumbling starts from one of the created penton vacancies and advances across the capsid until its complete demolition without following any pattern otherwise related to the virus geometry. This effect may be caused by the DNA escaping from the initial crack, and helping to demolish the viral shell as water enlarges an initially small crack of a dam wall. Conversely, immature capsids endure shorter times after penton release (40 ± 10% of the disruption time). Since *ts1* virions are less prone to release pentons, we infer that the energy provided by fatigue is used not only to remove vertex capsomers, but also to further weaken the rest of the viral shell. Penton vacancies in *ts1* seed the simultaneous formation of multiple cracks (Fig. 5b and S6). These cavities spread and coalesce roughly following the icosahedron edges, while the DNA remains condensed in the core. Thus, the extra shell-core connections in *ts1* keep the hexons attached to the core and preclude the wall crumbling that occurs in mature virions. Topographies of immature particles after disassembly (Fig. 3b, d) show intact core (red) plus dispersed hexons (black), but not DNA debris. The homogenous height decrease of 16 nm occurring in *ts1* particles (Fig. 3c, inset chart) corresponds to the viral wall thickness, and all final *ts1* structures exhibit heights of about 70 nm, which most likely corresponds to the remaining lower shell plus the intact core. These observations suggest that the viral shell has been torn away, while the exposed core remains stable. Precursor proteins tightly condense DNA inside *ts1* particles and keep it attached to the capsid walls, preventing genome release when the virus cage is removed (Fig. 3c, cartoon)^10^.

In conclusion, we present mechanical fatigue as a new route to induce single virus particle disruption while simultaneously monitoring the process. Fatigue disruption not only recapitulates the adenovirus disassembly pathway previously characterized by bulk or static imaging methods, but also enables for the first time a real time characterization of the intermediate dismantling stages of individual virions. Further, mechanical fatigue disassembly reflects the uncoating differences between mature and immature virions, providing a new way to characterize the role of maturation as a determinant of viral infectivity. One remarkable observation from the present study is that a source of stress so far removed from the *in vivo* situation as mechanical fatigue results in the same disassembly pattern induced by cellular cues. It brings to mind the idea that the disassembly program of each virus is deeply embodied in its architecture to guarantee successful infection. A prediction following this consideration is that bacteriophage capsids subject to controlled mechanical fatigue would not fall apart, but rather release their contents while conserving most of their structural organization as in their natural environment. Disruption by mechanical fatigue may therefore shed new light on the disassembly pattern of viruses for which no *in vivo* information is available on that regard.

## Methods

### Atomic force microscopy of adenovirus

Preparations of human adenovirus type 5 Ad5GL vector (used here as wildtype, mature virus) and immature *ts1* mutant virus were obtained as described^10^ and stored at −70°C in HBS buffer (20 mM Hepes, 150 mM NaCl, pH 7.8) in single-use aliquots. For AFM imaging, virus samples were diluted in a solution of NiCl_2_ in HBS to obtain a final solution of 5 mM of Ni^2+^ and virus concentrations between 1.5 – 2 × 10^12^ viral particles per ml. A drop of 20 μL of virus solution was deposited on freshly cleaved mica and incubated for 30 minutes at 4°C before washing with 5 mM NiCl_2_ in HBS. We do not use fixation agents such as glutaraldehyde because they provide additional mechanical strengthening^14^ that would impair virus disruption. The AFM tip was pre-wetted, and the mica was placed on a holder and immersed in 500 μL of the same buffer. The virus is therefore maintained in a hydrated, close to physiological state throughout the experiment. The AFM (Nanotec Electrónica S.L., Madrid, Spain) was operated in jumping mode in liquid^30^ with the modifications described in^31^, using RC800PSA (Olympus, Tokyo, Japan) cantilevers with nominal spring constants of 0.05 N/m. Cantilevers were routinely calibrated using Sader's method^41^. Disruption induced by mechanical fatigue strongly depends on the number of load cycles and the stress of each one, as predicted by the Whöler curve^32^. In order to gain temporal resolution and optimize the experiments as much as possible, we used the lowest force needed for obtaining stable AFM images and pixel density providing enough topographical resolution for virus particles. Images of 128 × 128 points and 300 × 300 nm^2^ were recorded scanning from left to right by applying forces of ~100 pN at a temperature of 20°C to avoid thermal drift. Inspection after the experiments revealed that the volume of the droplet (500 μl) remained essentially constant. Monitoring virus disassembly in real time was accomplished by repeatedly imaging each particle as described above. To build the disassembly movies, all images corresponding to the same virion were aligned. As a control that disruption was caused solely by mechanical stress and not by changes in environmental conditions during the prolonged imaging time, we observed that at the end of the experiment capsids in the neighborhood of the disassembled virion were still intact (Fig. S2). Capsid disruption events were analyzed in terms of elapsed time since acquisition of the first image, or loading cycles received, for each individual virion. Although obviously the times at which disassembly events happen strongly depends on the imaging conditions used, their relative value in our experimental setup illustrates the dynamics of adenovirus disassembly. TCDMs were constructed by subtracting each movie frame from the initial one for each virus. To highlight the lost shell area in each image, the difference maps were binarized by setting a gray level of 1 within the removed material zone and white level of 0 elsewhere. All the TCDMs for one individual virion were superimposed to obtain an image in which the grayscale levels indicate the sequence in which different areas of the shell were removed: the darker the pixel, the sooner this shell region was lost.

### Estimation of penton release energy

We took the maximum imaging force (100 pN) for each point of the virus surface, and the whole viral capsid is considered to have the same elastic response. The total energy provided to the virus during the forward cycle of a force-curve performed at a pixel in Jumping Mode is the enclosed area between the indentation curve and the x axis (Fig. S5). By using the spring constants of 0.38 N/m and 0.46 N/m for immature and mature virions^10^ respectively, the supplied energy per pixel is about 3.2 k_B_T (immature) and 2.6 k_B_T (mature) respectively (Fig. S5a). These values are likely overestimated, because part of the energy delivered by the forward curve can be given back due to the elastic response of the viral capsid, among other energy dissipating processes within the virus structure. A precise calculation of the energy would require the acquisition of both forward and backward curves. However, recording these data would slow down the speed of imaging and would affect real time monitoring. Still, for the sake of comparison between mature and immature particles, the energy provided to a virus while acquiring an image of 128 × 128 pixels can be estimated by taking into account all pixels, i.e. the force-distance curves, within the area delimited by the perimeter of the virus (Fig. S5b). The total energy is computed by multiplying the energy provided by one image by the number of images preceding the penton release. The energy required to cause penton release of WT and *ts1* virions is obtained by averaging the energies consecutively provided to create the penton vacancies of the triangular facet facing the tip. (Fig. 4c and S5c).

To compare with values obtained from a bulk technique, the energy required to cause the first disassembly events in WT or *ts1* capsids was estimated from the enthalpy corresponding to the first thermal transition in previously reported differential scanning calorimetry curves^10^, stated in terms of single virions instead of moles of hexon as before.

## Author Contributions

Alvaro Ortega-Esteban performed the experiments. A. Pérez-Berná, R. Menéndez-Conejero and S. J. Flint prepared the sample. C. San Martín and P. J. de Pablo conceived the study, coordinated the project and wrote the manuscript.

## Supplementary Material

Supplementary Information Supplementary Info

Supplementary Information Wild type (mature) virion disassembly

Supplementary Information Wild type (mature) virion disassembly

Supplementary Information ts1(immature) virion disassembly

Supplementary Information ts1 (immature) virion disassembly

Supplementary Information Wild type (mature) virion disassembly
