# Supplementary material for: Monitoring dynamics of human adenovirus disassembly induced by mechanical fatigue
Source: Sci Rep. 2013 Mar 13;3:1434. doi: 10.1038/srep01434 (PMC3595926; doi:10.1038/srep01434)
Supplement: Supplementary Information — Supplementary Info [file srep01434-s1.doc]

**Supplementary Information**

**Monitoring dynamics of human adenovirus disassembly induced by mechanical fatigue**

A. Ortega-Esteban^1^, A. J. Pérez-Berná^2^, R. Menéndez-Conejero^2^, S. J. Flint^3^, C. San Martín^2^, P. J. de Pablo^1^*

*[p.j.depablo@uam.es](mailto:p.j.depablo@uam.es)

^1^ Departamento de Física de la Materia Condensada, Universidad Autónoma de Madrid, 28049 Madrid (Spain)

^2^Department of Macromolecular Structure, Centro Nacional de Biotecnología (CNB-CSIC). Darwin 3, 28049 Madrid (Spain)

*^3^*Department of Molecular Biology, Princeton University, Princeton, NJ 08544, USA

*Rupture patterns obtained by single nanoindentation assays*

Classical nanoindentation assays consisted of exerting a force exceeding that of breakage ^1^ at the top of the particle (figure S1b). Afterwards an image of the virus was taken to confirm its disruption (figure S1c). For each virus analyzed, a breakage map corresponding to the force-distance curve (figure S1d) was computed by subtracting the final image after the nanoindentation from the intact virion image. The difference image was binarized to a gray level of 1 within the cracking area (figure S1e). After aligning all the virus images in the dataset using cross-correlation procedures in Xmipp^2^, breakage patterns were overlaid to obtain the average breakage pattern due to the nanoindentations (figure S1f and S1g). These breakage patterns do not provide evident differences between WT and immature virions.

***Supplementary figures***

**Figure S1**

Standard single nanoindentation experiments using large forces. (a) AFM image showing an intact adenovirus virion. (b) Example of a nanoindentation force-distance curve. Black and red are forward and backward cycles, respectively. (c) Final state of the virion showing the crack produced by the nanoindentation performed close to the center of the facet. (d) Difference map resulting from the subtraction of image (c) from (a). (e) Binarization of (d). (f and g) are average breakage maps of 9 WT and 12 *ts1* virions, respectively. Darker gray levels indicate higher frequency of breakage at these regions. These analyses show that viruses are mainly broken at the center, because the tip is meant to perform the indentation at the center of the triangular facet.

**Figure S2**

Virus disruption is due to mechanical fatigue, and not to the prolonged experimental time. (a) Two WT virions attached to the surface before the disassembly experiment. (b) A set of different stages throughout the disassembly of the lower viral particle are shown. The typical crumbling pattern for WT virus is observed. Frame numbers are indicated in each image. (c) The same area as in (a) imaged after the end of the disassembly experiment. The neighboring particle at the top remains intact.

**Figure S3**

(a) Adenovirus dilation ^3^ simulations performed on EM data before (a1) and after (a2) penton removal (a3 and a4 respectively) by using a 12 nm diameter tip. (a5) Comparison of profiles at the penton region (dotted lines). Figures (b3) and (b4) show the experimental AFM data, which are high-pass filtered in (b1) and (b2), respectively, to enhance penton vacancies. The profiles of the dotted lines are depicted at (b5), showing the same topography predicted in simulations. (c) Profiles demonstrate an excellent agreement between the theoretical and the experimental data of the penton vacancies.

**Figure S4**

Mechanical disruption recapitulates the temporal pathway of adenovirus uncoating in the cell. Virions escape the endosome 15 min p.i., having released some pentons. They reach the nuclear envelope at 45 min p.i. and are completely dismantled by 60 min p.i .In mature virions, the partially disrupted structure persists for 50-75% of the disassembly elapsed time which can be compared to the 66±8% due to mechanical fatigue disassembly.

**Figure S5**

Procedure to estimate the energy supplied for each penton release event. (a) Schematics showing the calculation of supplied energy for the scanning force in one point for WT (black) and *ts1* (red) viruses. (b) Area within the virus perimeter and number of pixels, i.e. number of force-distance curves in one image. (c) Supplied energies for each penton release event averaged to all ts1 and WT virions vacancies occurrences, indicated as E_1_, E_2_ and E_3_ for WT.

**Figure S6**

Time cumulative disruption maps (TCDMs). Examples of two-dimensional rupture maps for WT (a-c) and *ts1* (d-f) virions. The elapsed time in minutes corresponding to the whole grayscale for each image is 104 (a), 81 (b), 174(c), 104 (d), 90 (e), 92 (f), respectively. The scale bar corresponds to 45 nanometers in (a), 38 in (b), 46 in (c), 45 in (d), 49 in (e), and 50 in (f). Red contour plots indicate areas removed at 73% (a), 45% (b), 49% (c), 33% (d), 38% (e), 34% (f), of the total elapsed time, respectively. Green contour plots indicate areas removed at 90% (a), 75% (b), 75% (c), 73% (d), 65% (e), 79% (f), of the total elapsed time, respectively. Blue contour plots indicate areas removed at 96% (a), 94% (b), 92% (c), 80% (d), 73% (e), 93% (f), of the total elapsed time respectively. See also movies S1, S2, S3, S4 and S5.

**Table S1**

Average and mean statistical error of the cooperative factors n of height decrease during disassembly.

**Table S2**

Frames in which appearance of penton vacancies was observed, used for penton release distribution estimation shown in figure 3f. Data pertain to more particles than those included at figure 3a,b because some viruses were detached from the surface just after losing their pentons.

**Table S3**

Pentonless time expressed in % of the total disassembly time for each virion.

**Movies S1, S2 and S5**

Mechanical fatigue disruption of two different Wild Type (mature) virions.

First, a three-dimensional rendered topography of the virus is shown. Afterwards, the same sequence is shown stopping at clue points of the disassembly (7 frames per second). Lastly, a video of time cumulative disruption maps in which the breakage dynamics can be seen is shown (~5 frames per second). The time cumulative disruption maps (TCDMs) of figures S4c and S4b are obtained at the end of the movies, respectively.

**Movies S3 and S4**

Mechanical fatigue of two different *ts1* (immature) virions.

First, a three-dimensional rendered topography of the virus is shown. Afterwards, the same sequence is shown stopping at clue points of the disassembly (7 frames per second). Lastly, a video of time cumulative disruption maps is shown in wchich the breakage dynamics can be seen (~5 frames per second). The time cumulative disruption maps (TCDMs) of figures 4b and S4f are obtained at the end of the movies, respectively.

***Supporting references:***

^1^ Pérez-Berná, A. J. et al., The role of capsid maturation on adenovirus priming for sequential uncoating. *J. Biol. Chem.* **287** (37), 31582 (2012).

^2^ Scheres, S. H. W. et al., Image processing for electron microscopy single-particle analysis using XMIPP. *Nat. Protoc.* **3** (6), 977 (2008).

^3^ Villarrubia, J. S., Algorithms for scanned probe microscope image simulation, surface reconstruction, and tip estimation. *Journal of Research of the National Institute of Standards and Technology* **102** (4), 425 (1997).
